# When trust is threatened: Qualitative study of parents' perspectives on problematic clinical relationships in child cancer care

**DOI:** 10.1002/pon.4454

**Published:** 2017-06-08

**Authors:** Sarah Davies, Peter Salmon, Bridget Young

**Affiliations:** ^1^ Department of Psychological Sciences University of Liverpool Liverpool UK

**Keywords:** cancer, childhood leukaemia, clinical communication, oncology, parent‐clinician relationship, qualitative research

## Abstract

**Objective:**

We explored parents' accounts of the parent‐clinician relationship in childhood cancer to understand how parents who perceive threats to the relationship can be supported.

**Methods:**

Multicentre longitudinal qualitative study, with 67 UK parents of children (aged 1‐12 years) receiving treatment for acute lymphoblastic leukaemia. Analyses drew on the wider sample but focussed on 50 semistructured interviews with 20 parents and were informed by constant comparison.

**Results:**

All 20 parents described problems with clinical care such as inadequate information or mistakes by staff but varied in how much the problems threatened their sense of relationship with clinicians. Some parents saw the problems as having no relevance to the parent‐clinician relationship. Others saw the problems as threats to the clinical relationship but worked to “contain” the threat in ways that preserved a trusting relationship with at least one senior clinician. Parents' containment work protected the security they needed from the parent‐clinician relationship, but containment was a tenuous process for some. A few parents were unable to contain the problems at all; lacking trust in clinicians, these parents suffered considerably.

**Conclusions:**

Given the complexity of childhood cancer care, problems with clinical care are inevitable. By engaging in containment work, parents met their needs to feel secure in the face of these problems, but the extent to which parents should have to do this work is debatable. Parents could benefit from support to seek help when problems arise which threaten their trust in clinicians. Attachment theory can guide clinicians in giving this support.

## 1 INTRODUCTION

Good parent‐clinician relationships are important when a child undergoes cancer treatment.^1^, ^2^, ^3^ As the main providers of comfort and security,^4^ parents have an important role in their child's care and adjustment following diagnosis.^5^, ^6^ Alongside this role, parents will be managing their own fears about their child's survival.^7^ While some may benefit from specialist psychosocial interventions to alleviate their distress, routine clinical care is the mainstay of parental support following diagnosis of childhood cancer.^8^ We previously described how the care that clinicians provide as a routine part of treatment is profoundly important in comforting parents and helping them remain hopeful.^9^, ^10^ However, we also found that some parents perceived poor relationships with the clinicians caring for their child and described feeling unsupported.

Little is currently known about how to help such parents. Previous studies focussing on problems in the parent‐clinician relationship in childhood cancer have identified communication as an aspect of clinical care that can cause problems for parents. In the research literature, clinical communication difficulties have predominantly been conceptualised as failings by clinicians, linked to factors such as perceived medical errors, paternalism, and lack of empathy.^11^, ^12^, ^13^, ^14^ However, relationships reflect what each party brings.^15^ How parents perceive the clinician is likely, to some extent, to serve their own needs for comfort and security in the face of fears about their child.^16^ For example, in describing their reliance on clinicians, parents contrasted their feelings of panic and devastation at their child's diagnosis of cancer with the clinicians' expertise and calmness.^10^ Nevertheless, different parents or patients will have somewhat different needs and therefore might perceive the same clinician—and the clinician's care and communication—differently from others.^17^, ^18^, ^19^ Despite this, the role of parental factors in understanding problems in the parent‐clinician relationship has been little investigated.^16^ The development of interventions to promote better relationships is likely to depend on understanding the role of both parties in the relationship. Therefore, to complement research that has examined clinician factors, a commensurate focus on parental factors is warranted.

In areas that have received little investigation, quantitative studies can be premature because they risk imposing researchers' preconceptions. So we took an inductive approach, analysing qualitative interviews with parents who described pervasive difficulties in their relationships with clinicians to understand their needs and identify ways of helping them.

## 2 METHODS

### 2.1 Design

The data came from the RAPPORT study, a longitudinal qualitative study examining parents' relationships with the clinicians treating their children for acute lymphoblastic leukaemia.^9^, ^10^ Led by 2 of the current authors (BY and PS), data were collected between 2006 and 2009 in 3 phases: phase 1, approximately 37 weeks postdiagnosis; phase 2, 6 months; and phase 3, 12 months postdiagnosis. At each phase, one routine clinic consultation with the child's lead medical clinician was audio‐recorded and a researcher subsequently interviewed parents. Given our focus on exploring problems from the perspective of parents, analyses here draw only on the interviews.

### 2.2 Participants

RAPPORT gained UK NHS ethics approval (reference 06/MRE08/18). Consent was sought from parents of children aged 1 to 12 years. Children were receiving treatment at 6 principal paediatric oncology centres, representing 30% of such centres in the United Kingdom. We deemed families as ineligible if clinicians thought the parents were unable to give informed consent, the child had serious complications or was under the care of a nonparticipating clinician. Of 67 invited families, 43 (64%) participated (40 mothers, 27 fathers). This wider dataset has been described previously.^9^, ^10^ Given our current focus, we purposively sampled parents from this wider dataset that were “information rich”^20^ in that they described difficulties in their relationships with clinicians. We reviewed summary information about each family, written as part of earlier analyses, selecting parents who, in their interviews, had described serious and pervasive questions or doubts about care for their child that threatened parents' sense of relationship with any of the doctors or nurses. That is, parents described the problems as diminishing their sense of clinicians' competence or caring. We termed this the “threatened relationship” group. Additionally, we identified a “comparison group” of parents who had not reported such difficulties. We examined interviews with both groups in detail, comparing how parents in each described clinical care and relationships to delineate the ways in which the threatened relationship group was distinctive.

### 2.3 Procedure

In RAPPORT, a member of the health care team sought parents' permission for a qualitative researcher to contact them. The interviewers were a sociologist and an anthropologist with experience in qualitative research. They gave parents written information about the study and obtained consent. Face‐to‐face topic‐guided interviews were semistructured and conversational. Questions explored parents' perceptions of clinical care, their relationship with clinicians, and how the illness had affected the child and family. Mothers and fathers were usually interviewed separately in their homes. Audio‐recordings of interviews were transcribed, checked, and anonymised.

### 2.4 Analysis

Analysis was pluralistic, drawing upon thematic, interpretive, and narrative approaches to develop a contextualised understanding.^21^^.^ 22 Interviews were wide ranging, so analysis necessarily focussed on segments relevant to clinical relationships and interactions. This went beyond line‐by‐line coding of content, to attend to how participants' talked, including the particular words used, whether parents returned repeatedly to a topic, and to consider data in the context of the wider interview and successive interviews for each parent.

Initially, analysis was descriptive, focussing first on parents in the threatened relationship group and their experience of problematic interactions with members of the health care team. We worked inductively, progressively developing analytic categories by comparing interview data for each parent across phases (ie, within cases), as well as across parents (ie, between cases).^23^ We wrote narrative summaries of each case to explicate and develop the categories. Analysis of the comparison group data was initially informed by questions that had arisen from analysis of the threatened relationship group. However, we progressively iterated between both groups with our evolving questions and theoretical ideas in mind. Procedurally, analysis drew on the constant comparative method. We stopped sampling cases for the comparison group when new cases ceased contributing to the analysis. One author (SD), who became involved in the study in 2012, primarily performed the analysis. The others, who were familiar with the data from previous analyses, contributed through further review and discussion of transcripts and by commenting on draft reports of the analysis containing extensive data extracts. All 3 authors were psychologists, and 2 were clinically qualified.

In illustrative extracts from transcripts, we indicate treatment centres (A‐F), mother (M), father (F), clinician (C), (…) omitted speech, and [text] explanatory text.

## 3 RESULTS

### 3.1 Participant characteristics

We analysed 50 interviews with 20 parents. Table S1 summarises participant characteristics (see Supporting Information available online). Interviews lasted 30 to 232 minutes, and all except one parent were interviewed 2 or 3 times. The threatened relationship group comprised 12 parents from 4 centres, while the comparison group comprised 8 parents from 5 centres. Below, we first summarise findings from the threatened relationship group before turning to the comparison group.

### 3.2 Parents in the threatened relationship group

#### 3.2.1 All described problems related to clinical care, clinical interactions, or care “systems”

Throughout their interviews, these parents elaborated on numerous problems in their child's clinical care and, in interactions with clinicians, repeatedly describing how the problems left them frustrated. No parent had voiced these problems to the clinicians involved, although they had occasionally raised them with other staff. Parents in this group also described problems with the health care system, such as difficulties when children were transferred to nonspecialist wards due to bed shortages and appointment systems that resulted in long waits in clinics. Table S2 summarises the range of problems described.

#### 3.2.2 Parents described problems as threats to their relationships with clinicians

All parents in this group referred to how problems with care, interactions, or systems threatened their trust in clinicians' competence or care. As they explained, “It can give you serious doubts as to the care they're getting, as I say, when things are misprescribed” (D/F1); “She [doctor] doesn't answer your questions … you don't put your 100% trust in her … I am not so keen when I've got to see [her]” (F/M6). When parents perceived that incompetence had caused their child unnecessary distress, their responses were particularly intense: “I'd quite happily throttle that guy if I saw him again” (E/F1). As well as questioning clinicians' competence, these parents also doubted their motives. For example, parents wondered if clinicians sometimes deliberately withheld information: “I felt like I was being kept in the dark” (D/F1) and therefore avoided parents: “It's like they're trying to avoid you because you want to know … avoid the question whatsoever … and that's frustrating” (F/M3) and described feeling that clinicians did not value them: “You feel like you're an inconvenience … I felt like as if they weren't interested almost” (F/M3); “You're just another number passing through for a day” (A/F8). While the problems these parents described with the health care systems were not directly related to parents' interactions with clinicians, they reduced parents' confidence in the care they were receiving.

Throughout their interviews 2 parents repeatedly described these problems as encompassing all clinical relationships and as destroying their trust in clinicians. One (F/F3) explicitly described distrusting clinicians' competence. The other (A/M1) described distrusting their intentions; that is, she did not feel clinicians cared. Table S3 illustrates how these perceptions pervaded these parents' accounts of their relationships with clinicians, including those leading their child's care. In both cases, the perceptions endured over time; indeed, A/M1's perceptions of clinicians worsened.

In contrast, the remaining 10 parents in this group indicated that they had preserved their trust in one or more of their child's clinicians, particularly the senior clinicians. In describing how the problems affected them, these parents displayed a range of strategies: deciding to trust the expertise of the lead clinicians; attributing problems to pressures on clinicians; focussing on clinical interactions that had been positive; blaming the system that clinicians worked in; and accepting the problems as inevitable (Table S4). These allowed parents to separate the problems from a global sense of clinicians' competence and caring motives, thereby isolating the threat and protecting the security parents needed from the clinical relationship. We term this process “containment.” Referring to clinicians, one parent spoke explicitly of containment work: “parents have to make a decision whether to reinforce or deconstruct what's going on, and all we can do is reinforce it because it's the best thing we've got” (E/F1), indicating his conscious decision to maintain trust in clinicians despite the problems and his awareness of both the tenuousness and necessity of containment. Although these parents varied in the extent of mistrust and containment that they reported, unlike the 2 parents above (F/F3 and A/M1), all 10 described at least one senior clinician whose competence and intentions they did not doubt or question. Indeed, several reflected in their later interviews on how their sense of relationship with these clinicians had deepened: “as time's gone on and you get to know him a bit better, he's quite, um, approachable … that's what you need” (D/M9) or transformed over time:

Christ, I wouldn't have said this 12 months ago. I think [Doctor C2] is an absolutely fantastic person … there's certain people in life that you'll never forget and I think now, looking at it over the 12 months, he is one of them (F/F5).

#### 3.2.3 Parents in the comparison group also experienced problems with clinical care, but these did not threaten clinical relationships

We examined the interview transcripts of the 8 comparison group parents to identify whether the sorts of problems that parents in the threatened relationship group described were specific to those parents. All comparison group parents reported at least one problem related to clinical care and most reported several. The types of problems described by parents in the 2 groups were indistinguishable; for instance, both described problems with information and communication, long waits, clinical mistakes, and lack of adaptation to children's needs. However, unlike parents in the threatened relationship group, comparison group parents either did not perceive the problems as threats to their relationships with clinicians, or they quickly dispelled such threats. Thus, what differentiated the accounts of the 2 groups was not whether parents reported problems in clinical care, but how they made sense of the problems. Whereas parents in the threatened relationship group saw problems as signs to doubt clinicians, or even not to trust them at all, parents in the comparison group construed the problems as having little or no relevance to their sense of relationship with clinicians. That is, parents in the comparison group engaged in containment, as did most in the threatened relationships group, but comparison group parents differed in containing all the problems they experienced.

We illustrate the “complete containment” of parents in the comparison group with a typical case from this group (Table S5). While the problems this father discussed were similar to those described by parents in the threatened relationship group, he mentioned each problem only once, and there was no evidence that these threatened his perception of clinicians as competent or caring. In his final interview, this father described positive and trusting relationships with clinicians: “They … are really good … I've had no … qualms with them at all … in the whole year” (A/F7). Other parents from the comparison group explicitly reflected on initial problems that had arisen early in treatment. At phase 2, a father explained that he did not “jump up and down” about problems because “these people are here in our case to save, you know, a child from dying … and everything else is peripheral” (D/F3). At phase 3, another referred to earlier problems with the communication style of his clinician, yet now described this clinician as “our saviour” and “absolutely brilliant” (B/F6).

## 4 DISCUSSION

All parents described problems related to clinical care, the health care system, or their interactions with clinicians. These problems were similar to those described in previous studies of parents' experience of children's cancer care, in which the problems were seen as the source of difficulties in the parent‐clinician relationship.^11^, ^12^, ^13^, ^14^, ^24^, ^25^ However, our analysis went beyond this, showing that the problems parents reported were not what marked them out as perceiving difficulty in relationships with clinicians. All parents, including those who perceived wholly positive and trusting clinical relationships, described problems related to clinical care. What distinguished parents who reported threatened relationships with clinicians was their reactions to those problems. For parents in the threatened relationship group, the problems led them to doubt the competence and caring intentions of some clinicians, and 2 parents profoundly distrusted all their clinicians. In contrast, no parents in the comparison group described the problems in this way.

The accounts of the comparison group parents and most in the threatened relationship group illustrate the extent to which parents can work to contain challenges to their relationships with clinicians and to actively manage threats to these relationships. We described a range of containment strategies, such as focussing attention on interactions with clinicians that were positive and blaming “the system” rather than clinicians. The findings resemble elements of attribution theory, which explains contrasting perceptions of similar social behaviours in terms of whether the causes that individuals assign are internal (eg, personality characteristics) or external (eg, situational factors).^26^ However, most parents in the threatened relationship group made both types of attribution, and this theory offered little to explain their motivation for making one or other type. We therefore turned to attachment theory, which has been extended from its origins as a theory of the earliest human relationships^4^ to understand how, in times of considerable threat like severe illness, adults form relationships with authority figures such as clinicians to help them feel secure.^27^, ^28^, ^29^, ^30^ Drawing on research that has described how adult cancer patients construct their clinicians as “attachment figures,”^31^^,^ 32 we propose that parents' containment work functioned to isolate problems related to clinical care from their perception of the clinical relationships surrounding it. That is, parents' containment work made sense of threats in ways that protected their mental image of at least one of their clinicians as a secure base. Containment thereby met the needs of parents to feel safe in the care of someone they regarded as having the expertise to do what was needed for their child^27^ and so protected parents from being overwhelmed by their fears. For parents in the threatened relationship group, however, containment was a tenuous or incomplete process, and their doubts or concerns about clinicians were often not contained. Moreover, 2 parents in this group distrusted all clinicians and did not engage in containment work at all. Feeling unable to rely on any of their clinicians, they suffered considerably.

As previous studies of the parent‐clinician relationship and communication in childhood cancer have indicated,^11^, ^12^, ^13^, ^14^, ^17^, ^24^, ^25^ the ways in which clinicians respond to parents is important in understanding the relationship. However, the relationship will also be influenced by what parents bring to interactions with clinicians and how they interpret events, yet previous studies have rarely investigated these.^16^ In investigating such influences, we have drawn attention to the work that parents do to create and sustain the sort of clinical relationships that they need to feel safe. In showing that parents differ in their tendency to contain threats to their sense of relationship with clinicians, our analysis is consistent with research indicating that people vary in their ability to trust clinicians.^28^, ^33^, ^34^

While previous studies of problematic parent‐clinician relationships in childhood cancer have tended to rely on samples recruited from parent support groups,^16^ a strength of our study is that parents were recruited from several UK paediatric oncology centres. Few families attend support groups,^34^ so samples drawn from such groups likely provide limited insights. Moreover, by drawing on a comparison group of parents who did not perceive difficulties in clinical relationships, our analyses illuminated the processes giving rise to these perceived difficulties. Additionally, our inductive approach allowed us to understand these difficulties from the perspectives of parents. Theory can strengthen qualitative research,^35^ and as noted above, attachment theory informed our interpretations. We and others have previously found this theory helpful for understanding clinical relationships^27^, ^28^, ^29^, ^30^, ^31^, ^32^, ^36^ in which, like those we studied, one of the parties is vulnerable. Attachment theory drew our attention to parents' need to feel safe as motivating their perceptions of clinicians in the face of the problems they described. Nevertheless, we acknowledge that other interpretations are possible.

### 4.1 Study limitations

The study also has limitations. First, while all 3 authors were involved in the analysis, we did not independently categorise the 2 groups and we may have missed some threatened relationship cases in the wider sample. However, the study's aim was not to provide generalisable estimates of the number of parents perceiving threats to their clinical relationships but rather to illuminate processes that give rise to such difficulties. Secondly, the data were collected over 7 years ago. With the continued emphasis on family centred clinical care, it is possible that parents now experience fewer problems, although there is little evidence for this.^37^ Moreover, the relational processes we describe are likely to resist changes at the policy level alone.^38^

### 4.2 Clinical implications

In considering the implications for practice, it is important to acknowledge that no clinician, relationship, or system of care can be perfect. In the complex and profoundly emotional context of childhood cancer care, it is inevitable that parents will experience problems, as all did in our analyses. For some parents, these problems threatened their trust in clinicians. Considered in the light of attachment theory, our findings point to ways that clinicians could understand and support such parents. A parent's profound distress and fears for their child activate their own attachment needs, and although the parent will work to make sense of problems in ways that protect their perceptions of clinicians as attachment figures, this is a tenuous process. Some parents will, at times, struggle to trust clinicians and feel safe, and a few may never trust or feel safe. We acknowledge that care practices and systems can fail, and in such cases, parents' lack of trust is a signal for improvement.^39^ However, the clinical communication literature is dominated by work that reaches such conclusions. In contrast, there is little literature on the processes we have identified and on how the problems parents describe will sometimes indicate their need to *feel* better cared for, rather than necessarily an objective problem that can be eradicated.

Future research is needed to investigate clinicians' perspectives on how to address this need. One approach could be for clinicians to discuss, at an early stage in treatment, the sorts of concerns and doubts that parents can experience during their child's treatment, and to explain that clinicians are open to addressing such concerns with parents if they arise, and occasionally revisiting these discussions. In the absence of such discussions, the likelihood is that parents will continue to suffer quietly, as parents in our study did, and that clinical care that threatens parental trust will endure, hidden from clinicians by the containment work that most parents use to protect their sense of relationships with them. Moreover, while there was no indication that the relationship difficulties we studied had escalated into overt conflict, discussions of this sort may help to prevent the disintegration of relationships^40^ when severe problems arise.

### 4.3 Conclusion

In conclusion, we found that all parents in these analyses encountered several problems with clinical care, the health care system, and in interactions with clinicians. However, it was not problems per se that marked parents out as perceiving difficulties in their relationships with clinicians. The findings raise wider questions about the extent to which a parent's sense of clinical relationships should be reliant on the work they do to contain problems. Support that helps parents to *feel* better cared may help to reduce this reliance. Future research could investigate clinicians' perspectives on how to support parents who perceive clinical relationship difficulties and how clinicians could help them to feel safe in their care despite parents' profound fears.

## CONFLICT OF INTEREST

The authors have declared that there is no conflict of interest.

## Supporting information

Table S1 Participant characteristics

Table S2 Problems with parent‐clinician interactions and systems reported by parents in the ‘threatened relationship’ group

Table S3 Distrusting clinicians' competence (F/F3) and distrusting clinicians' caring intentions (A/M1)

Table S4 Ways that parents protected the security of the parent‐clinician relationship

Table S5 Typical case indicating that parents in the comparison group perceived similar problems as parents in the ‘threatened relationship’ group

## References

[1] Gibbins J , Steinhardt K , Beinart H . A systematic review of qualitative studies exploring the experience of parents whose child is diagnosed and treated for cancer. J Pediatr Oncol Nurs. 2012;29:253‐271. 22907681 10.1177/1043454212452791

[2] Wolfe J , Klar N , Grier HE , et al. Understanding of prognosis among parents of children who died of cancer: impact on treatment goals and integration of palliative care. JAMA. 2000;284:2469‐2475. 11074776 10.1001/jama.284.19.2469

[3] Young B , Dixon‐Woods M , Findlay M , et al. Parenting in a crisis: conceptualising mothers of children with cancer. Soc Sci Med. 2002;55:1835‐1847. 12383468 10.1016/s0277-9536(01)00318-5

[4] Bowlby J . *Attachment and Loss: Attachment* (vol. 1). New York, Basic Books; 1969.

[5] Fedele DA , Hullmann SE , Chaffin M , et al. Impact of a parent‐based interdisciplinary intervention for mothers on adjustment in children newly diagnosed with cancer. J Pediatr Psychol. 2013;38:531‐540. 23471362 10.1093/jpepsy/jst010 PMC3666119

[6] Pierce L , Hocking MC , Schwartz LA , Alderfer MA , Kazak AE , Barakat LP . Caregiver distress and patient health‐related quality of life: psychosocial screening during pediatric cancer treatment. Psychooncology. 2016; https://doi.org/10.1002/pon.4171 10.1002/pon.4171 27321897

[7] Long KA , Marsland AL . Family adjustment to childhood cancer: a systematic review. Clin Child Fam Psychol Rev. 2011;14:57‐88. 21221783 10.1007/s10567-010-0082-z

[8] Hocking MC , Kazak AE , Schneider S , Barkman D , Barakat LP , Deatrick JA . Parent perspectives on family‐based psychosocial interventions in pediatric cancer: a mixed‐methods approach. Support Care Cancer. 2014;22:1287‐1294. 24337762 10.1007/s00520-013-2083-1 PMC3969770

[9] Salmon P , Hill J , Ward J , Gravenhorst K , Eden T , Young B . Faith and protection: the construction of hope by parents of children with leukemia and their oncologists. Oncologist. 2012;17:398‐404. 22371382 10.1634/theoncologist.2011-0308 PMC3316926

[10] Young B , Hill J , Gravenhorst K , Ward J , Eden T , Salmon P . Is communication guidance mistaken? Qualitative study of parent–oncologist communication in childhood cancer. Brit J Cancer. 2013;109:836‐843. 23900218 10.1038/bjc.2013.413 PMC3749579

[11] Clarke JN . Essential work for mothers of children living with cancer. J Psychosoc Oncol. 2006;24:31‐47. 17046805 10.1300/J077v24n02_03

[12] Clarke JN , Fletcher P . Communication issues faced by parents who have a child diagnosed with cancer. J Pediatr Oncol Nurs. 2003;20:175‐191. 14567565 10.1177/1043454203254040

[13] Moore JB , Kordick MF . Sources of conflict between families and health care professionals. J Pediatr Oncol Nurs. 2006;23:82‐91. 16476782 10.1177/1043454205285871

[14] Ringnér A , Jansson L , Graneheim UH . Parental experiences of information within pediatric oncology. J Pediatr Oncol Nurs. 2011;28:244‐251. 21653912 10.1177/1043454211409587

[15] Hargie O , Dickson D . Skilled Interpersonal Communication: Research, Theory and Practice. 4th Edition ed. London: Routledge; 2004.

[16] Davies S , Young B , Salmon P . Towards understanding problems in the parent‐practitioner relationship when a child has cancer: meta‐synthesis of the qualitative literature. Psychooncology. 2016; https://doi.org/10.1002/pon.4285 10.1002/pon.4285 27642695

[17] Hummelinck A , Pollock K . Parents' information needs about the treatment of their chronically ill child: a qualitative study. Patient Educ Couns. 2006;62:228‐234. 16139981 10.1016/j.pec.2005.07.006

[18] Thorne S , Armstrong E‐A , Harris SR , et al. Patient real‐time and 12‐month retrospective perceptions of difficult communications in the cancer diagnostic period. Qual Health Res. 2009;19:1383‐1394. 19805801 10.1177/1049732309348382

[19] Thorne SE , Kuo M , Armstrong EA , McPherson G , Harris SR , Hislop TG . ‘Being known’: patients' perspectives of the dynamics of human connection in cancer care. Psychooncology. 2005;14:887‐898. 16200520 10.1002/pon.945

[20] Patton M . Qualitative Research and Evaluation Methods. London: Sage; 2002.

[21] Salmon P , Mendick N , Young B . Integrative qualitative communication analysis of consultation and patient and practitioner perspectives: towards a theory of authentic caring in clinical relationships. Patient Educ Couns. 2011;82:448‐454. 21111558 10.1016/j.pec.2010.10.017

[22] Clarke NJ , Willis ME , Barnes JS , et al. Analytical pluralism in qualitative research: a meta‐study. Qual Res Psychol. 2015;12:182‐201.

[23] Miles MB , Huberman AM . Qualitative Data Analysis: An Expanded Sourcebook. New York: Sage; 1994.

[24] Lozowski S , Chesler MA , Chesney BK . Parental intervention in the medical case of children with cancer. J Psychosoc Oncol. 1994;11:63‐88.

[25] Moore JB , Beckwitt AE . Parents' reactions to conflict with health care providers. West J Nurs Res. 2003;25:30‐44. 12584962 10.1177/0193945902238834

[26] Watson D . The actor and the observer: how are their perceptions of causality divergent? Psychol Bull. 1982;92:682

[27] Salmon P , Young B . Dependence and caring in clinical communication: the relevance of attachment and other theories. Patient Educ Couns. 2009;74:331‐338. 19157761 10.1016/j.pec.2008.12.011 PMC3764431

[28] Holwerda N , Sanderman R , Pool G , et al. Do patients trust their physician? The role of attachment style in the patient‐physician relationship within one year after a cancer diagnosis. Acta Oncol. 2013;52:110‐117. 23113593 10.3109/0284186X.2012.689856

[29] Ciechanowski PS . As fundamental as nouns and verbs? Towards an integration of attachment theory in medical training. Med Educ. 2010;44:122 20653845 10.1111/j.1365-2923.2009.03578.x

[30] Tan A , Zimmermann C , Rodin G . Interpersonal processes in palliative care: an attachment perspective on the patient‐clinician relationship. Palliat Med. 2005;19:143‐150. 15810754 10.1191/0269216305pm994oa

[31] Salander P . Bad news from the patient's perspective: an analysis of the written narratives of newly diagnosed cancer patients. Soc Sci Med. 2002;55:721‐732. 12190266 10.1016/s0277-9536(01)00198-8

[32] Wright EB , Holcombe C , Salmon P . Doctors' communication of trust, care, and respect in breast cancer: qualitative study. BMJ. 2004;328:864 15054034 10.1136/bmj.38046.771308.7C PMC387476

[33] Pegman S , Beesley H , Holcombe C , Mendick N , Salmon P . Patients' sense of relationship with breast cancer surgeons: the relative importance of surgeon and patient variability and the influence of patients' attachment style. Patient Educ Couns. 2011;83:125‐128. 20684871 10.1016/j.pec.2010.04.023

[34] Harding R , Beesley H , Holcombe C , Fisher J , Salmon P . Are patient–nurse relationships in breast cancer linked to adult attachment style? JAN. 2015;71:2305‐2314. 10.1111/jan.12693 26037680

[35] Reeves S , Albert M , Kuper A , Hodges BD . Why use theories in qualitative research. BMJ. 2008;337:631‐634. 10.1136/bmj.a949 18687730

[36] Maunder RG , Hunter JJ . Can patients be ‘attached’ to healthcare providers? An observational study to measure attachment phenomena in patient–provider relationships. BMJ Open. 2016;6:e011068. 10.1136/bmjopen-2016-011068 PMC4874120 27178976

[37] Shields L , Zhou H , Pratt J , Taylor M , Hunter J , Pascoe E . Family‐centred care for hospitalised children aged 0‐12 years. Cochrane Libr. 2012;10:CD004811. 10.1002/14651858.CD004811.pub3 PMC11531909 23076908

[38] Pilnick A , Dingwall R . On the remarkable persistence of asymmetry in doctor/patient interaction: a critical review. Soc Sci Med. 2011;72:1374‐1382. 21454003 10.1016/j.socscimed.2011.02.033

[39] Gallagher TH , Mazor KM . Taking complaints seriously: using the patient safety lens. BMJ Qual Saf. 2015;24:352‐355. 10.1136/bmjqs-2015-004337 25977314

[40] Forbat L , Teuten B , Barclay S . Conflict escalation in paediatric services: findings from a qualitative study. Arch Dis Child. 2015;100:769‐773. 25940425 10.1136/archdischild-2014-307780 PMC4518764

