# Supplementary material for: When trust is threatened: Qualitative study of parents' perspectives on problematic clinical relationships in child cancer care
Source: Psychooncology. 2017 Jun 8;26(9):1301–6. doi: 10.1002/pon.4454 (PMC5600008; doi:10.1002/pon.4454)
Supplement: Supplementary file 1 — Table S1 Participant characteristics [file PON-26-1301-s001.docx]

**Table S.1 Participant characteristics**

| **ID** | **Group** | **Mother or father** | **Age range of child** | **Occupational status** |
| --- | --- | --- | --- | --- |
| A/M1 | Threatened relationship | Mother | 10-12 | Skilled/ Manual |
| A/M2 | Threatened relationship | Mother | 1-4 | Professional/ Managerial |
| A/F6 | Threatened relationship | Father | 1-4 | Skilled/ Manual |
| A/F8 | Threatened relationship | Father | 5-9 | Professional/ Managerial |
| D/F1 | Threatened relationship | Father | 1-4 | Professional/ Managerial |
| D/F9 | Threatened relationship | Father | 5-9 | Professional/ Managerial |
| D/M9 | Threatened relationship | Mother | 5-9 | Professional/ Managerial |
| E/F1 | Threatened relationship | Father | 5-9 | Professional/ Managerial |
| F/F3 | Threatened relationship | Father | 1-4 | Skilled/ Manual |
| F/M3 | Threatened relationship | Mother | 1-4 | Professional/ Managerial |
| F/F5 | Threatened relationship | Father | 1-4 | Professional/ Managerial |
| F/M6 | Threatened relationship | Mother | 1-4 | Professional/ Managerial |
| A/M7 | Comparison | Mother | 1-4 | Professional/ Managerial |
| A/F7 | Comparison | Father | 1-4 | Professional/ Managerial |
| B/F2 | Comparison | Father | 5-9 | Professional/ Managerial |
| B/F6 | Comparison | Father | 1-4 | Skilled/ Manual |
| C/F3 | Comparison | Father | 5-9 | Professional/ Managerial |
| D/F3 | Comparison | Father | 5-9 | Skilled/ Manual |
| D/M11 | Comparison | Mother | 1-4 | Skilled/ Manual |
| F/M2 | Comparison | Mother | 5-9 | Professional/ Managerial |
