# Supplementary material for: When trust is threatened: Qualitative study of parents' perspectives on problematic clinical relationships in child cancer care
Source: Psychooncology. 2017 Jun 8;26(9):1301–6. doi: 10.1002/pon.4454 (PMC5600008; doi:10.1002/pon.4454)
Supplement: Supplementary file 2 — Table S2 Problems with parent‐clinician interactions and systems reported by parents in the ‘threatened relationship’ group [file PON-26-1301-s002.docx]

**Table S.2 Problems with parent-clinician interactions and systems reported by parents in the ‘threatened relationship’ group**

| - **Lack of time or opportunities in consultations to ask questions or clarify understanding**   “*He’d asked if there had been any questions but it’s like, “Any questions” you know, in his quick manner, “Right I need the next person”* (A/M2).  “*This sounds really stupid after a year but we still don't totally understand some of this stuff*.” (E/F1).   - **Poor communication between clinicians**   “*It’s almost like we have to take responsibility for bridging the communications* [between staff] *and letting each other, the other side know*.” (A/F8).  “*One of the biggest problems is communication between the staff*.” (D/F1).   - **Long waits**   “*It just depends when you turn up to go and see the clinic… You could be there for hours just sitting there waiting.”* (A/F6).  “*You’re there sort of three or four hours, she’s* [my child] *had nothing to eat.”* (D/F1).   - **Noticing apparent mistakes in medical care**   *“When you’re going to the nurses saying… I know the dose of steroids she has, this isn’t right*.” (D/F1).  “*Junior doctors and the like coming down was my, a particular annoyance of mine. So at one point she had she’d had about four cannulas in various places and a junior doctor came down and and had three unsuccessful attempts, which ruined all the veins in one hand…It was just like having to put up with people who are worse at certain things*.” (E/F1).   - **Being placed on non-oncology wards**   “*It was a ward down in the hospital* [outside oncology] *…And it wasn't very nice, you know*.” (A/M1).   - **Clinicians not adapting practice for individual children**   “*The frustrating part is people being pigheaded about the way they do it and we had one nurse… who insisted, ‘Out your bed. Up to there and get a shower…and basically dragged [my child] along kicking and screaming and scrubbed her down and washed everything… but I know that kid. She just needs to be persuaded”* (E/F1). |
| --- |
