# Supplementary material for: When trust is threatened: Qualitative study of parents' perspectives on problematic clinical relationships in child cancer care
Source: Psychooncology. 2017 Jun 8;26(9):1301–6. doi: 10.1002/pon.4454 (PMC5600008; doi:10.1002/pon.4454)
Supplement: Supplementary file 3 — Table S3 Distrusting clinicians' competence (F/F3) and distrusting clinicians' caring intentions (A/M1) [file PON-26-1301-s003.docx]

**Table S.3 Distrusting clinicians’ competence (F/F3) and distrusting clinicians’ caring intentions (A/M1)**

| **Distrusting clinicians’ competence (F/F3)**  In his phase one interview F/F3 fleetingly made some remarks that implied clinicians were not to blame for the problems he experienced: “*I mean nothing’s sort of intentional or anything, I think, these things happen*” However, as the interview progressed he described mistakes that he perceived clinicians had made on several occasions and his doubts about their competence became more evident: “*Was it that one that just wasn’t feeling well, so they were forgetting what they were doing? You just don’t know. And then obviously you’re starting to think about it.”*  By phase two he was more certain that the mistakes he perceived indicated the incompetence of clinicians: “*It is hectic at times, but they seem in no rush about anything… But it just seems like they forget*” “*I'd say some of the nurses, you know, they're hopeless*.” He described how the mistakes he perceived led him to accept training to complete some medical tasks for his child at home, despite some initial reluctance to take on these tasks. Similarly, in hospital he began to closely monitor treatment and seek involvement in medical tasks: “*I said, "Do you know when she's getting cytarabine? Because," I said, "I'm trained. I can do it" … So this stupid nurse from other ward number, who I think they've just dragged her off the street, you know, she hasn't got a clue about anything. Then a doctor actually did it … and the way we've been taught is put saline in, chemo, saline, heprin. He put chemo straight in. I'm sure he did. I asked the nurse after and she said, "Oh, you maybe never saw right." I thought, I did.”* Referring to the training he had received for administering these medicines this father added “*And I said, "Well, I thought I was doing this, anyway*”  At phase three the father’s vigilance continued, although he did not report any further mistakes by clinicians: “*I mentioned in the last interview, it was about when she was having cytarabine when the doctors didn't do it right … we're sort of eagle eyed … just to make sure nothing's amiss, you know, but err, no I don't think there's been anything, anything else really*.” |
| --- |
| **Distrusting clinicians’ caring intentions (A/M1)**  A/M1 believed strongly in the benefits of alternative medicine and worried about the impact on her child of the drugs used in medical treatment. At phase one she felt unable to ask the questions about her child’s treatment that she needed to: “*I still don't know and that was something I wanted to ask Dr C2 but I just couldn't… I don't feel relaxed.* ”  By phase two A/M1 struggled to believe that clinicians were being truthful with her. For instance she thought that a change in her state benefit payment might indicate that her child’s prognosis had worsened and that she had not been informed about this: “*I thought, why is [my son] getting high rate- higher rate care? … and I thought well most people who I knew who got that, like had a terminal illness… so you start thinking well is that the hospital's way of telling me? And is my son terminally ill*?”  At phase three A/M1’s relationships with clinicians had further deteriorated and she now spoke of how clinicians were purposely withholding information: “*Because, by them withholding that knowledge from me*” She described feeling hurt by the apparent indifference of clinicians: “*I feel like I'm there and like everyone's around you but no one's like taking any notice*.” Similarly, her account of when her child had his central line removed (a surgically inserted tube used to administer chemotherapy), a significant milestone as it marks the end of the intensive treatment phase, illustrated her sense that staff were avoiding her and her child: “*This is us, you know, like a celebration. But then no one really came over and spoke to us or nothing … because then, what I feel then is, I feel then that people are staying away from me, you see? … I sat next to my son’s bed and started, I was crying*.” |
