# Supplementary material for: When trust is threatened: Qualitative study of parents' perspectives on problematic clinical relationships in child cancer care
Source: Psychooncology. 2017 Jun 8;26(9):1301–6. doi: 10.1002/pon.4454 (PMC5600008; doi:10.1002/pon.4454)
Supplement: Supplementary file 4 — Table S4 Ways that parents protected the security of the parent‐clinician relationship [file PON-26-1301-s004.docx]

**Table S.4 Ways that parents protected the security of the parent-clinician relationship**

| **Trusting in the expertise of lead clinicians**  All nine parents in the ‘threatened relationship’ described pervasive threats to clinical relationships, yet trusted in the expertise of at least one of their child’s clinicians and spoke positively of the skills and knowledge of these clinicians. Often parents described having made a decision to trust a particular clinician at their very first meeting, as D/F1 explained: “*You sort of make your mind up whether you’ve got confidence in this person. He seemed very confident in what he was doing, in his knowledge.*” Parents described these trusted clinicians as knowing best and acting in parents’ interests. For instance, in subsequent interviews one parent reframed his initial frustration with a lack of information from a clinician as a belief that this clinician was withholding information for the benefit of parents: “*Giving us the information in increments… bit like, you know, baiting, I was a little bit annoyed but, but now I realise just exactly why… our ability to cope would have been impeded with, if they’d just given us a whole snapshot*.” (E/F1).  **Attributing problems to the pressures on clinicians**  Parents frequently described clinicians as busy and overloaded and gave this as the reason for the problems that they encountered rather than the neglectful or poor behaviour of a clinician. For example, D/M9 described her frustration after numerous failed attempts to put questions to her child’s clinician, whilst attributing the clinician’s lack of response to the pressure he was under: “*they have had quite a few new cases last week… So they have been very, very busy… it’s just that I’ve got some questions about it [child’s treatment plan]… so it has been a bit frustrating really that I’ve not managed to pin him down*.”  **Focussing on interactions with clinicians that were positive**  Parents emphasised times that they felt cared for and valued by clinicians. F/M3 discussed the first time that clinicians called her by name: “*It just makes you feel that you're somebody. And that you matter and that you're a person. You know, it just made a huge difference to me.*” Parents discussed feeling confident in the positive intentions of clinicians because they felt they had come to know them: *“It just looks like they're doing nothing … I know they're not like that at all*” (A/F6).  **Blaming “the system”**  Parents also avoided blaming some clinicians by invoking a system that was beyond the control of the staff within it: “*The NHS* UK National Health Service *is this grinding, rumbling machine, which just rumbles along at its own pace. And you jump on and jump off, and jump, but you cannot control it, you know, even to the staff*” (E/F1).  **Accepting the problems as inevitable**  Instead of blaming clinicians parents accepted the problems as *“just one of those things*” (F/F5) or “*just part of hospital life”* (A/M2). Mistakes were seen as understandable and inevitable: “*They're human, aren't they*?” (A/F6). |
| --- |
