# Supplementary material for: When trust is threatened: Qualitative study of parents' perspectives on problematic clinical relationships in child cancer care
Source: Psychooncology. 2017 Jun 8;26(9):1301–6. doi: 10.1002/pon.4454 (PMC5600008; doi:10.1002/pon.4454)
Supplement: Supplementary file 5 — Table S5 Typical case indicating that parents in the comparison group perceived similar problems as parents in the ‘threatened relationship’ group [file PON-26-1301-s005.docx]

**Table S.5 Typical case indicating that parents in the comparison group perceived similar problems as parents in the ‘threatened relationship’ group**

| A/F7 encountered difficulties that parents in the ‘threatened relationship’ group commonly described, including long waits: “*They say, "Bring him in…within an hour. Start him on the antibiotics. "Sets off, got there about five o'clock and, er, there wasn't enough nurses on the in-patients ward. …So then they take us up to the oncology ward and basically we were hanging round there till half ten at night, just in the corridors.”*  Mistakes with bloods test led to inconvenience and frustration that added to his child’s suffering: *“I had to go back for bloods. That annoyed me … they can’t find the results and you have to go back. Or they took one sample and not two and she had to go back.”*  The father described the problems that arose when his child was cared for outside the oncology wards: *“But with them little cubicles, because it's so narrow and you've got, like, rooms going right the way down, you just feel like isolated.”*  He also described junior clinicians struggling to insert a cannula correctly: “*My son doesn’t like the needles going in anyway. The clinician was like trying to get it in and faffing about and … then my son is well upset isn’t he. Eventually they had to bring in a more senior doctor but I know they’ve got to train I know that but when it’s your child going through it you’re just thinking ‘just leave him alone because I know you’re not going to get it, why not just bring in someone?’* ”  A/F7 appeared to accept these problems with minimal distress: *“The days that you’ve got to be there all day you know you’ve got to be there at all day so you, you’re sort of expecting it.”* The problems did not affect his relationship with clinicians. Indeed, he spoke positively of the nurses: *“Can't fault them”* and the doctors*: “They know best, don't they? …You know that your kid's getting the best treatment…and they're getting well looked after.”* |
| --- |
